# The relationship among adiponectin, high sensitive C reactive protein and triacylglycerol level in healthy young persons

**DOI:** 10.1186/1476-511X-10-109

**Published:** 2011-06-30

**Authors:** Juxiang Li, Chenghong Liao, Hai Su, Qiang Peng, Zhihong Zhang, Sujian Yan, Qing Yang

**Affiliations:** 1 Department of Cardiology, Second Affiliated Hospital, Medical College of Nanchang University. 330006, NO 1 Mingde Road, Nanchang, Jiangxi, PR of China; 2 The Fuzhou Medical College of Nanchang University, Fuzhou, Jiangxi, PR of China; 3 The Research Institute of Cardiovascular Disease of Nanchang University. 330006, NO 1 Mingde Road, Nanchang, Jiangxi, PR of China

## Abstract

**Background:**

The elevated postprandial triacylglycerol (TG) concentration is associated with elevated coronary artery disease. Oral fatty tolerant test (OFTT) is less performed in the health. This study was to evaluate the effect of sex and body mass index (BMI) on postprandial TG concentration of the low fat meal in healthy young persons. This study included 112 healthy college students (18.8+/-1.6y). Their body height and weight were measured for body mass index (BMI). According to BMI, 27 subjects were in the under-weight subgroup, 60 in the normal weight subgroup and 25 in the over-weight subgroup. After overnight fasting low fat OFTT (27 g fat, 600 kcal) was performed and the plasma TG and glucose concentrations were measured before and at 2, 4 and 6 hour after a fat meal. The area under the curve (AUC) of TG was calculated.

**Results:**

The fasting TG levels were similar and the fasting TG levels gradually increased as BMI increased in both sexes. The postprandial TG levels at 2 and 4 h decreased in female, but did not significant change in male. In female, the TG curves of 3 BMI subgroups showed saddle type, but in male the TG curve of the over-weight subgroup had a peak at 2 h, on the other hand the TG curve of under- weight subgroup had a dip at 2 h.

**Conclusions:**

Gender and BMI are important influencing factors for TG metabolism after fat meal in the youth. The young male persons with over-BMI have abnormal TG metabolism.

## 

The elevated fasting and postprandial plasma triacylglycerol (TG) levels are risk factors for cardiovascular disease and type 2 diabetes [1-3]. As postprandial TG has been considered as a more important risk than fasting TG level, oral fatty tolerant test (OFTT) is often used to identify postprandial TG metabolic abnormality. Some studies demonstrated that age, gender and race play a significant role in the TG metabolic profiles among individuals [4]. As obesity and over-weight have become an important healthy problem and the prevalence of atherosclerosis related diseases increase gradually in the youth, early intervention of fasting and postprandial TG abnormality becomes an urgent task. However, OFTT is performed often in middle-aged or elderly population, but seldom in the youth, especially in the healthy youth.

Recently, many studies have demonstrated that adiponectin can modulate a number of metabolic processes, including glucose regulation and fatty acid catabolism [5,6]. Adiponectin may associate with the postprandial TG in OFTT [7]. As a general marker for inflammation and infection, high sensitive C reactive protein (hsCRP) is associated with an increased risk of diabetes, hypertension and cardiovascular disease [8]. Recently, the relation of OFTT with adiponectin and hsCRP is concerned, but litter is known in the youth. Is the TG response to OFTT different from that in middle-aged or elderly population in the youth? What relationship of adiponectin and hsCRP with OFTT response exists in the youth? This study was designed to evaluate the postprandial TG response to a fat meal and the role of sex and body mass in 112 healthy young college students.

## Subjects and methods

### Subjects

In this study, 112 healthy, normolipidemic students including 41 men and 71 women from a medical college (18.8 ± 1.6 y) were enrolled. On the morning of the test day, their blood pressure (BP), heart rate (HR), body height and weight were measured, and body mass index (BMI) was calculated. According to the BMI standards of WHO for Asians, the students were divided into three subgroups: the under-weight subgroup included 27 subjects (M = 7 , F = 2 0), the normal weight subgroup included 60 (M = 19 , F = 41) and the over-weight subgroup included 25 (M = 15, F = 10) students. The ages of three BMI subgroups were similar.

This study was approved by the Ethic Committee of the Second Affiliated Hospital of Nanchang University and the subjects offered informed consent.

## Methods

### Fat meals

The low fat meal contains a dose of 27 g fat , 24 g protein and 67 g carbohydrate, with a total energy content of 600 kcal. The percentage of fat, protein and carbohydrate were 42%, 18% and 50% respectively.

### OFTT

The subjects fasted overnight for 12 h and did not drink alcohol on the test day. Before a cannula was inserted into a vein for blood sampling, the subjects had rested for 15 min, and the first blood sample was taken as baseline( 0 h or fasting ). Then a fat meal was ingested within 15 minutes. The repeated blood samples were drawn at 2, 4 and 6 hours after the fat meal again. In the following period all subjects were watching TV in order to avoid the effect of exercise and no food was allowed except drinking small amount of water.

### Parameters

The HR and BP in sitting position were taken 2 times and the average was used as the final values. The plasma triacylglycerol (TG) and glucose concentrations were measured with HITACHI 7600-020. The values of TG and glucose at 0 h, 2 h, 4 h and 6 h were used to calculate the area under the curve (AUC).

The fasting hsCRP was measured with a high-sensitivity latex-enhanced turbidimetric assay with Quantex CRP ultra sensitive kits supplied by BIOKIT USA. The fasting adiponectin was measured by Adiponectin ELISA kits (UAS).

### Statistical methods

All data are presented as Mean ± SE in the text and tables. SPSS10.0 statistical version( SPSS Company, Chicago, Illinois, USA )was used for the analysis of variation (ANOVA) and Student's t-test. Statistical significance was defined as P < 0.05.

## Results

### The general information

Table 1 shows the general information of the 112 students. The BP, HR and BMI were higher in the male group.

**Table 1:** The general information in the 112 healthy youth( x ± SE )

	total(n = 112)	M(n = 41)	F (n = 71)
SBP( mmHg )	104.03 ± 1.27	114.05 ± 2.17	98.24 ± 1.12*
DBP(mmHg)	66.98 ± 0.86	70.49 ± 1.48	64.96 ± 0.99*
HR(bpm)	76.51 ± 0.91	72.93 ± 1.36	78.69 ± 1.15*
BMI	21.21 ± 0.35	22.67 ± 0.70	20.374 ± 0.35

comparison with male , * P < 0.05

### The curves of TG and PG between sexes

**Figure 1:**
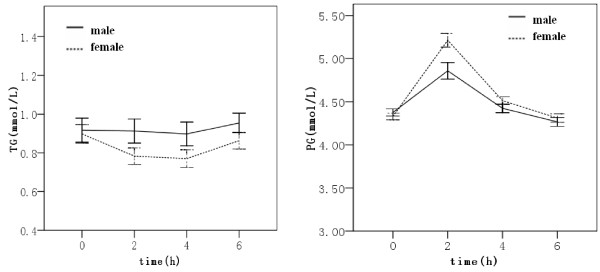
**The curves of TG and glucose in the male and female**. The left panel: TG curve; The right panel: glucose curve

The fasting TG concentration was similar between two sexes (Figure 1). The postprandial TG levels at the 2 and 4 hours after low fat meal were lower than fasting level, forming s saddle type curve. However, the TG curve of male is near to a horizontal line. The TG-AUC of female was lower than that of male( -0.52 ± 1.26 vs -0.009 ± 1.28 h•mmol/L, p < 0.05 ).

On the other hand, the blood glucose curve of the female had a higher peak at 2 h after fat meal and higher PG-AUC (2.11 ± 1.84 vs 0.95 ± 1.71 h•mmol/L, p < 0.01.) in female.

### The gender TG and glucose curves among 3 BMI subgroups

In the total group, the fasting TG levels gradually increased as BMI increased, so the 3 TG curves on BMI subgroups separated at the beginning. Typical saddle TG curves appeared in the under- and normal weight subgroups, even in the over-weight subgroup the postprandial TG level did not increase. However, the TG curves showed same difference between mala and female, in female, the curves of 3 BMI subgroups showed saddle type, but in male, the TG curve of the over-weight subgroup had a peak at 2h. These results indicated that gender and BMI are important factors for TG metabolism after fat meal.

**Figure 2:**
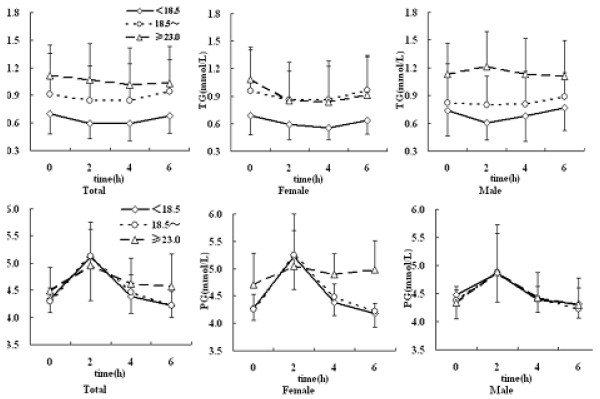
**The curves of TG and glucose among 3 BMI subgroups between sexes**. The upper panel: TG curve; The down panel: glucose curve

The profile of 3 blood glucose curves in 3 subgroups was similar in total group, but the over-weight subgroup showed higher levels at 0, 4 and 6 h and a lower peak at 2 h, and this character only occurred in female(Figure 2).

### The plasma adiponectin and hsCRP concentrations between sexes on BMI

**Figure 3:**
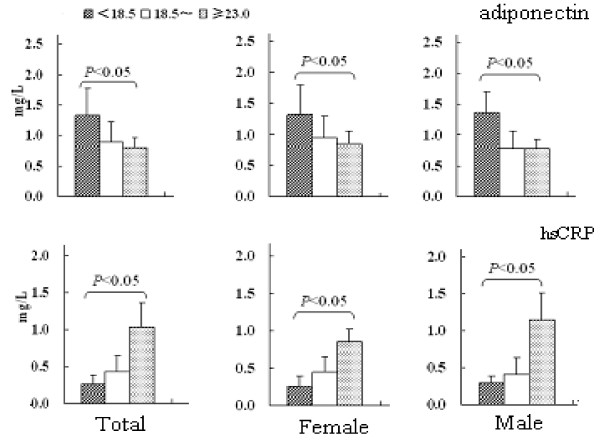
**The plasma adiponectin and hsCRP concentrations on BMI level**.

Figure 3 clearly shows that the plasma adiponectin concentrations decreased, but the plasma hsCRP concentrations increased as the BMI increased in total, or in male or female.

The plasma adiponectin concentrations were similar between male and female among 3 BMI subgroups. In the BMI > 23.0 subgroup, the hsCRP was higher in male than in female.

### Correlation analysis

The hsCRP levels related positively not only with TG0 (r = 0.315, p < 0.01), but with TG2 (r = 0.374, p < 0.01). No relation between adiponectin and TG0, but a negative relation was found between adiponectin and TG 2 (r = -0.210, p < 0.05). These results indicated that postprandial TG was stronger predictor for fasting hsCRP and adiponectin levels.

A negative relation existed between adiponectin and CRP (r = -0.433(p < 0.01) also. There is a positive relation of TG0 with TG 2 ( r = 0.794, p < 0.01).

## Discussion

More and more data indicate that fasting TG level is related to coronary heart disease. Meanwhile, the postprandial TG has been considered as a more important risk than fasting TG level [1]. So the increased postprandial TG becomes a target of intervention for preventing atherosclerosis -related diseases.

This study discovered a very interesting, but confusing finding, that is the postprandial plasma TG level after a fat meal was lower than fasting plasma TG level in female and low BMI male. On the common consideration the plasma TG level should increase, rather than decrease after a fat meal, even low fat meal. All of the previous papers showed increased postprandial plasma TG level and increment TG-AUC in OFTT, although the increase extent may be different [1-3,5]. Based on our knowledge, this is the first report discovering decreased postprandial TG level after a fat meal.

We suggested that the younger age, low BMI and low fat meal were the factors responsive for the decreased postprandial TG level. As the under- weight subgroup had the lowest fasting TG level and most obvious postprandial TG decline, and the over- weight male subgroup showed postprandial TG increases at 2 h after fat meal. At the same fat intake, the postprandial TG decline seen in the under- and normal weight subgroups may reflect that they have stronger TG metabolic capability, if not so, the postprandial TG should be higher than the over-weight subgroup. So, it is reasonable to suggest that even in the youth, the over-weight male group may have abnormal TG metabolism as comparison with normal weigh male group. Previous results showed also that the obesity and over-weight are favorable factors for impaired (over) reaction of postprandial TG in middle-age and elderly [6].

Adiponectin and CRP are associated with lipid metabolic abnormality. Adiponectin plays an important role in glucose and lipid metabolism. Some studies demonstrated that adiponectin can decrease plasma TG levels by increasing the TG and VLDL-TG catabolism by the way to increase skeletal muscle lipoprotein lipase (LPL) and VLDL receptor expression [9]. Although negative correlation between fasting adiponectin and triglycerides (TG) (r = -0.25 - -0.34) was found in postmenopausal females, in patients with coronary artery disease and in obese diabetic and non-diabetic Caucasians [10-12], our study did not show relationship between fasting adiponectin and fasting TG levels, the younger population in this study may be the reason for the different results.

However, our study did show that the adiponectin level related negatively with postprandial TG at 2 h after fat meal (r = -0.21, p < 0.05). These results suggest that comparison with fasting TG, postprandial TG may be more important for adiponectin level.

As a general marker for inflammation and infection, elevated CRP level is related with an increased risk of diabetes, hypertension and cardiovascular disease [8,13,14] Our result showed that a stronger positive relationship was found between hsCRP and TG2 (r = 0.374, p < 0.01) than between hsCRP level and TG0 (r = 0.315, p < 0.01), which suggests that high postprandial TG may be more important for high hsCRP levels than fasting TG. As TG2 level has stronger predicting capability for higher fasting hsCRP and adiponectin level then fasting TG level, OFTT is valuable for indentifying the fat metabolic abnormality.

Adiponectin has anti-inflammatory, anti-diabetic, and anti-atherogenic properties, on the other hand, the hsCRP is inflammatory factor. As several studies demonstrated a negative relationship of BMI with fasting adiponectin, and positive relationship with hsCRP [5-7], and negative relationship between adiponectin and hsCRP was demonstrated by our and others' studies [15], we can point out that even in young population, over-weight is an rick for the abnormal TG metabolism, high systemic inflammation and development of atherosclerotic disease. Regular exercise can prevent over-weight and induce low inflammatory state [16].

The limitation of this study is not to determine the TG at 1 hour or earlier after fat meal, so we could not fully discover the more earlier response of TG to fat meal and the mechanism for the decreased postprandial plasma TG level, as there may be a possibility that increased TG occurs at earlier stage after fat meal, and then the body switches on a regulation mechanism to prevent plasma TG rise, or even induce a lower TG level than fasting if the regulation mechanism is strong.

## Conclusion

Even in the youth, the over-weight males have abnormal TG metabolism and are in high systemic inflammatory statement. Comparison with the fasting TG, the postprandial TG at 2 hour is a more valuable predictor for the fasting adiponectin and hsCRP.

## Abbreviations

TG: triacylglycerol; OFTT: oral fatty tolerant test; hsCRP: high sensitive C-reactive protein; BP: blood pressure; HR: heart rate; BMI: body mass index; AUC: area under the curve

## Competing interests

The authors declare that they have no competing interests.

## Authors' contributions

Conduct of the study: JL, CL; QP

Design and manuscript writing: HS, SY, QY

Data collection and analysis: ZZ and QY

All authors have read and approved the final manuscript.

## Funding Sources

This study was supported by a grant from the Chinese National Science and Technology Plan (2008 BAI68B02).
